# Supplementary material for: Complexome profiling of the Chlamydomonas psb28 mutant reveals TEF5 as an early PSII assembly factor
Source: Plant Cell. 2025 Mar 17;37(6):koaf055. doi: 10.1093/plcell/koaf055 (PMC12164590; doi:10.1093/plcell/koaf055)
Supplement: koaf055_Supplementary_Data [file koaf055_supplementary_data.zip › psb28_interactive.zip!/plots/intensity/qualityPlots/peptides/ASD1_Cre09.g389689.t1.1.html]

### Cre09.g389689.t1.1

Trivial name: ASD1  
SeqCoverage [median]: 12.20  
Mass: 39.93 kDa  
  
 

| Sequence | MissedCleavage | Mass [Da] | wtIntensity | muIntensity | Score |
| --- | --- | --- | --- | --- | --- |
| AGAIVVDNSSAFR | 0 | 1305.67 | 0.00 | 1843.42 | 81.34 |
| DVLDGKPAQPK | 1 | 1166.63 | 341.07 | 1087.74 | 48.44 |
| MTEGVPLVIPEVNPEAMSHIK | 0 | 2290.17 | 0.00 | 976.12 | 50.96 |
|  |  |  | 341.1 | 3907.3 |  |
